# Can the Spatial Heterogeneity in the Epiligament Explain the Differential Healing Capacities of the ACL and MCL?

**DOI:** 10.3390/jcm15020510

**Published:** 2026-01-08

**Authors:** Lyubomir Gaydarski, Boycho Landzhov, Richard Shane Tubbs, Georgi P. Georgiev

**Affiliations:** 1 Department of Anatomy, Histology and Embryology, Medical University of Sofia, 1431 Sofia, Bulgaria; lgaidarsky@gmail.com (L.G.); landzhov_medac@abv.bg (B.L.); 2 Department of Anatomical Sciences, St. George’s University, St. George 1473, Grenada; shane.tubbs@icloud.com; 3 Department of Neurosurgery, Tulane University School of Medicine, New Orleans, LA 70112, USA; 4 Department of Neurology, Tulane University School of Medicine, New Orleans, LA 70112, USA; 5 Department of Structural and Cellular Biology, Tulane University School of Medicine, New Orleans, LA 70112, USA; 6 Department of Surgery, Tulane University School of Medicine, New Orleans, LA 70112, USA; 7 Department of Orthopedics and Traumatology, University Hospital Queen Giovanna-ISUL, Medical University of Sofia, 1527 Sofia, Bulgaria

**Keywords:** knee, lower limb, anterior cruciate ligament, medial collateral ligament, epiligament, CD34, α-SMA, VEGF, ligament healing, progenitor cells, myofibroblast, angiogenesis, surgery, anatomy

## Abstract

**Background:** The anterior cruciate ligament (ACL) and medial collateral ligament (MCL) display strikingly different healing behaviors, despite their similar structural roles within the knee. The epiligament (EL)—a vascular and cellular envelope surrounding each ligament—has emerged as a critical determinant of repair capacity. The aim of this study was to perform a region-specific, comparative analysis of EL molecular profiles in the ACL and MCL to elucidate the mechanisms underlying their contrasting reparative outcomes. **Methods:** Human ACL and MCL specimens were obtained from 12 fresh knee joints. Immunohistochemical labeling for CD34, α-smooth muscle actin (α-SMA), and vascular endothelial growth factor (VEGF) was performed across proximal, mid-substance, and distal EL regions. Quantitative image analysis using IHC Profiler for ImageJ generated semiquantitative (negative, low-positive, positive) distributions, and inter-ligament comparisons were quantified using *t*-tests (*p*  <  0.05). **Results:** Distinct, region-specific EL signatures were identified. The ACL EL exhibited strong proximal α-SMA expression (0% neg/66.8% low+/33.2%+) and notable distal CD34 positivity (0% neg/83.3% low+/16.7%+), while VEGF expression was confined to the mid-substance (≈55% low+/26%+). In contrast, the MCL EL was largely negative for CD34 and VEGF across all regions, showing a homogeneous but functionally oriented α-SMA profile: proximally negative, sparse mid positivity, and high distal low-positive staining (93.4% low+). Differences in proximal and distal CD34 and α-SMA expression between the ACL and MCL were highly significant (*p*  <  0.0001–0.001), confirming a mechanistic divergence in EL organization. **Conclusions:** The ACL EL is regionally heterogeneous, vascularly biased, and enriched in contractile α-SMA+ cells, suggesting localized but poorly coordinated reparative potential. In contrast, the MCL EL is structurally uniform, with distributed α-SMA activity supporting stable wound contraction and tissue continuity, despite limited angiogenic signaling. These findings indicate that the ACL’s failure to heal is not attributable to the absence of progenitor or angiogenic factors, but rather to its fragmented spatial organization and dominant contractile phenotype. Therapeutically, preserving and modulating the EL, particularly its CD34+ and α-SMA+ compartments, could be key to enhancing intrinsic ACL repair and improving outcomes in ligament reconstruction and regeneration.

## 1. Introduction

Knee ligament injuries are among the most common and functionally limiting problems encountered in sports medicine and orthopedics, producing pain, mechanical instability, and progressive joint degeneration [1,2]. The ACL and MCL are the ligaments most frequently involved in such injuries [3], the MCL being reported as the single most commonly injured knee ligament in some series [4,5]. Epidemiological estimates for MCL injury range from 0.24 to 7.3 per 1000 persons, with a male-to-female predominance of approximately 2:1 [6,7]. Typical MCL trauma results from a valgus load to the lateral knee, a mechanism frequently encountered in contact sports such as football and ice hockey [8,9].

The ACL is also commonly injured [10,11], with an estimated incidence of 68.6 cases per 100,000 population in the United States [12,13]. The ACL is most often ruptured during sporting activities that require twisting, cutting, or rapid changes in direction, and approximately 70% of ACL tears are non-contact injuries that occur during abrupt deceleration or pivoting maneuvers [12,14,15]. Importantly, ACL rupture is a well-recognized risk factor for the later development of osteoarthritis [16]. The rising incidence of ACL injury over time has therefore made it an increasing focus of orthopedic practice and research.

Despite the superficial similarities between the ACL and MCL, their healing trajectories differ markedly. The extra-articular location of the MCL confers a relatively strong capacity for spontaneous repair [9]. However, the reparative tissue that forms after MCL injury often exhibits biomechanical properties inferior to those of the native ligament [3,9]. In contrast, the intra-articular ACL demonstrates minimal spontaneous mid-substance healing. Following complete rupture, the ends of this ligament often retract and fail to establish a functional bridging scar, resulting in persistent instability that commonly necessitates surgical reconstruction for patients wishing to return to high-demand activities [17,18,19].

A recent reframing of ligament biology highlights the epiligament (EL), a thin, metabolically active connective tissue layer that envelops the ligament proper, as a potentially decisive factor in repair. Histologically distinct from the relatively hypocellular and hypovascular ligament core, the EL is highly cellular and vascular, acting as a local reservoir of reparative elements, including fibroblasts, progenitor cells, blood vessels, matrix metalloproteinases, and growth factors [3,18,19,20]. Cells and signaling molecules within the EL are proposed to migrate through the endoligament into the ligament body after injury, contributing to inflammation, matrix synthesis, neovascularization, and remodeling [3,18,19]. EL-derived fibroblasts synthesize multiple extracellular matrix constituents—collagen isoforms, fibromodulin, decorin, and fibronectin—that regulate degradation, proliferation, and structural reorganization during healing [3,18,19].

In the present study, this epiligament-centered hypothesis was tested by examining key molecular mediators of repair within the EL. We focused on three complementary markers: CD34, α-smooth muscle actin (α-SMA), and vascular endothelial growth factor (VEGF). CD34 is a transmembrane glycoprotein that characterizes hematopoietic stem cells and endothelial progenitor cells (EPCs). EPCs can home to injury sites and differentiate into endothelial cells, contributing to vasculogenesis, and a local population of CD34+ cells indicates progenitor availability for neovascularization and regeneration [21,22,23,24,25]. Preclinical studies further show that local delivery of CD34+ cells enhances MCL healing, primarily by promoting neovascularization and a pro-regenerative microenvironment [26,27,28,29,30,31].

α-SMA is an actin isoform that marks contractile cell phenotypes—vascular smooth muscle cells and myofibroblasts [32,33]. Myofibroblasts appear transiently during wound repair and express α-SMA de novo, assembling it into stress fibers that generate contractile force [33,34]. This contractility drives wound contraction and matrix remodeling, processes central to restoring tissue continuity and mechanical function [32,33,34].

VEGF is the principal paracrine mediator of angiogenesis, driving endothelial proliferation, migration, and tube formation in response to tissue hypoxia and inflammatory signals after injury [35,36,37]. Angiogenesis is a prerequisite for successful repair because newly formed vessels deliver oxygen, nutrients, and inflammatory and progenitor cells to the healing site and remove metabolic waste [35,36,37].

In our previous work, we assessed the regional expression of these markers in the EL of ACL and MCL separately [18,19,38,39]. We showed that CD34, α-SMA, and VEGF are present in the EL of both ligaments but with different marker- and region-specific patterns. Overall, these proteins are mostly located in vascular compartments of the EL, but they differ in their tissue distribution and prominence: α-SMA is enriched in vascular/superficial EL layers, consistent with a myofibroblastic/smooth-muscle signal; VEGF is concentrated in vessel walls; and CD34 marks the vascular-derived progenitor/endothelial populations in connective tissues, depending on the site. In the MCL EL, we observed relatively strong superficial α-SMA staining and a distal enrichment of cellularity while VEGF remained principally vascular. In contrast, our ACL studies highlighted prominent vascular-associated α-SMA together with segmental variation in CD34/VEGF signals. Together, those previous findings support the view that the EL supplies vascular, myofibroblastic, and progenitor elements that are likely to influence ligament biology and repair [18,19,38,39]. To our knowledge, no study has directly compared the regional expression of these markers in the EL of the ACL and MCL. A systematic, region-specific analysis could elucidate the molecular basis for their differing healing capacities and reveal distinct patterns of repair across ligament subregions.

The aim of the present study was to compare the histological morphology of the ACL and MCL, with particular emphasis on their EL. We quantified immunohistochemical expression of CD34, α-SMA, and VEGF in the proximal, mid-substance, and distal regions of each EL and integrated these morphological and molecular data into an EL-centered model to explain the ligaments’ differential, region-specific healing responses.

## 2. Materials and Methods

### 2.1. Human Tissue Samples

Tissue was obtained from 12 fresh human knee joints (five male, seven female; mean age ≈ 55 years at death) donated to the Department of Anatomy, Histology, and Embryology at the Medical University of Sofia. No donor had documented knee osteoarthritis, prior knee surgery or known traumatic knee injury.

### 2.2. Immunohistochemistry (IHC)

For IHC, 4 µm paraffin sections were deparaffinized, and endogenous peroxidase activity was blocked according to the manufacturer’s recommendations. The primary monoclonal antibodies used were anti-α-SMA (M0851, DAKO/Agilent, Santa Clara, CA, USA), anti-VEGF (M7273, DAKO/Agilent), and anti-CD34 (M7165, DAKO/Agilent), each applied at a 1:100 dilution. The EnVision™ FLEX+ (Mouse, High pH, K8002, Agilent, Santa Clara, CA, USA) polymer system was used for detection with 3,3′-diaminobenzidine (DAB) as chromogen; slides were counterstained with Mayer’s hematoxylin. Negative/technical controls were included (eighteen control sections reported across experiments). Photomicrographs of representative fields were captured with an Olympus CX21 microscope fitted with an Olympus C5050Z digital camera (Evident Corporation, Tokyo, Japan).

### 2.3. Image Acquisition and Semiquantitative Scoring

Whole-slide photomicrographs or representative fields were analyzed using ImageJ (NIH ImageJ, v1.53f51). We utilized 600 vision fields per group (12 knees; 5 slides per knee; 10 vision fields per slide). Immunostaining intensity was scored semi-automatically with the IHC Profiler plugin, which reports a four-tier category (high positive 3+, positive 2+, low positive 1+, negative 0). In the reported work, five slides per ligament were analyzed, and at least ten random visual fields per slide were scored. Final group scores were calculated as the average across fields/images. All followed a well-established protocol from our previous studies [18,38,39].

### 2.4. Statistical Analysis and Data Visualization

Image-derived numerical data were processed and visualized in R (R v4.x) using RStudio v2023.03.0 + 386 and ggplot2. Groups were compared using *t*-tests (or multiple *t*-tests with Holm–Sidak correction where multiple pairwise comparisons were required); a two-sided *p*  <  0.05 was considered statistically significant. All image-processing, scoring, and segmentation steps were performed blinded to experimental grouping where possible, and automated scoring (IHC Profiler) was used to reduce observer bias.

## 3. Results

**Figure 1:**
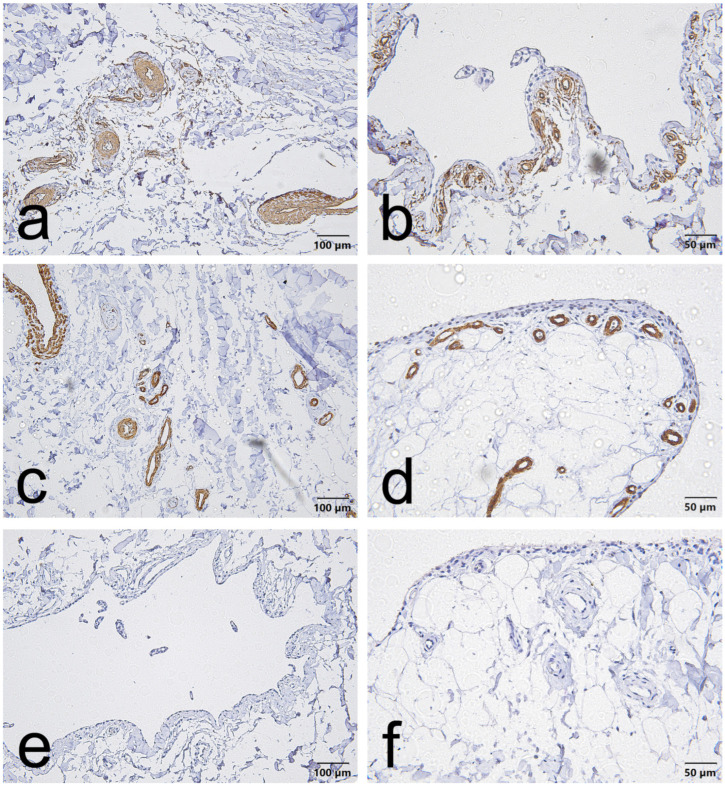
Representative immunohistochemistry of the proximal part of the ACL epiligament (EL). Panels show serial fields stained for CD34 (**a**,**b**), α–smooth muscle actin (α-SMA) (**c**,**d**), and vascular endothelial growth factor (VEGF) (**e**,**f**). Panels (**a**,**c**,**e**), scale bar = 100 µm; panels (**b**,**d**,**f**), scale bar = 50 µm.

**Figure 2:**
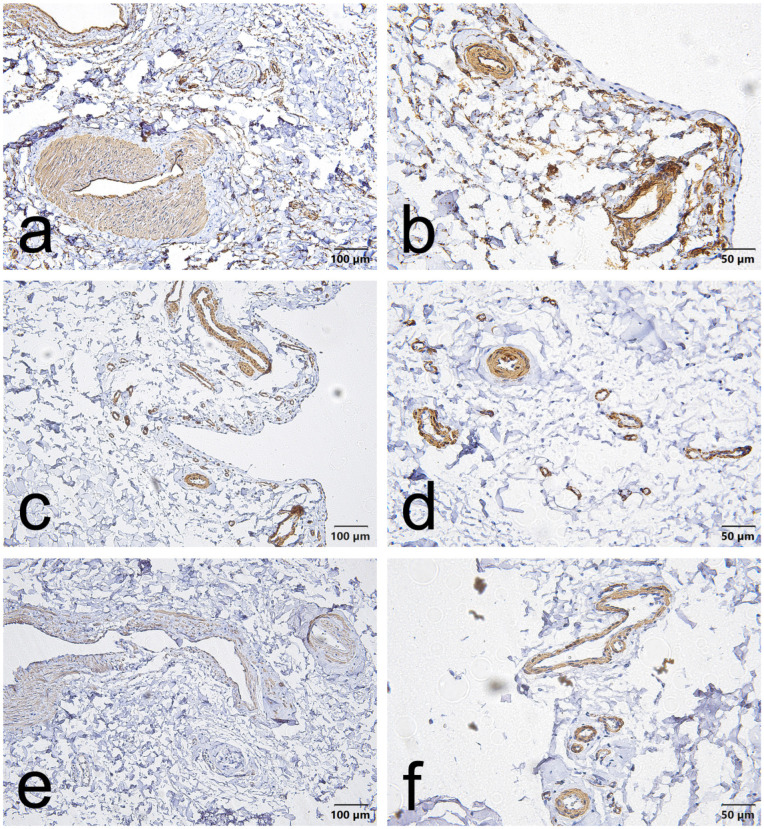
Representative immunohistochemistry of the mid-substance of the ACL epiligament (EL). Panels show serial fields stained for CD34 (**a**,**b**), α–smooth muscle actin (α-SMA) (**c**,**d**), and vascular endothelial growth factor (VEGF) (**e**,**f**). Panels (**a**,**c**,**e**), scale bar = 100 µm; panels (**b**,**d**,**f**), scale bar = 50 µm.

**Figure 3:**
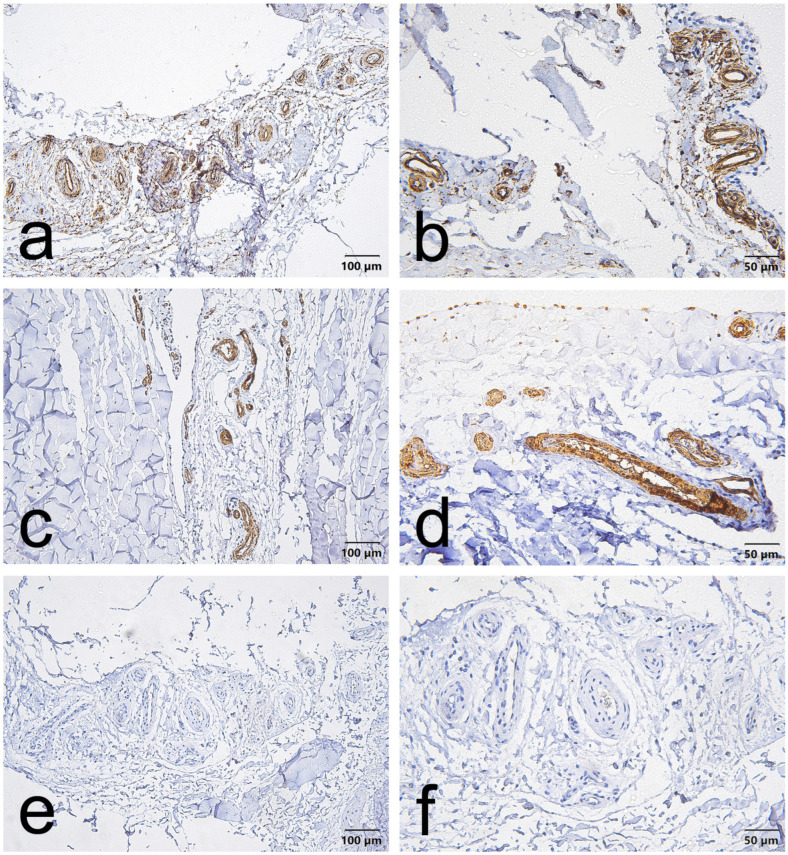
Representative immunohistochemistry of the distal part of the ACL epiligament (EL). Panels show serial fields stained for CD34 (**a**,**b**), α–smooth muscle actin (α-SMA) (**c**,**d**), and vascular endothelial growth factor (VEGF) (**e**,**f**). Panels (**a**,**c**,**e**), scale bar = 100 µm; panels (**b**,**d**,**f**), scale bar = 50 µm.

**Figure 4:**
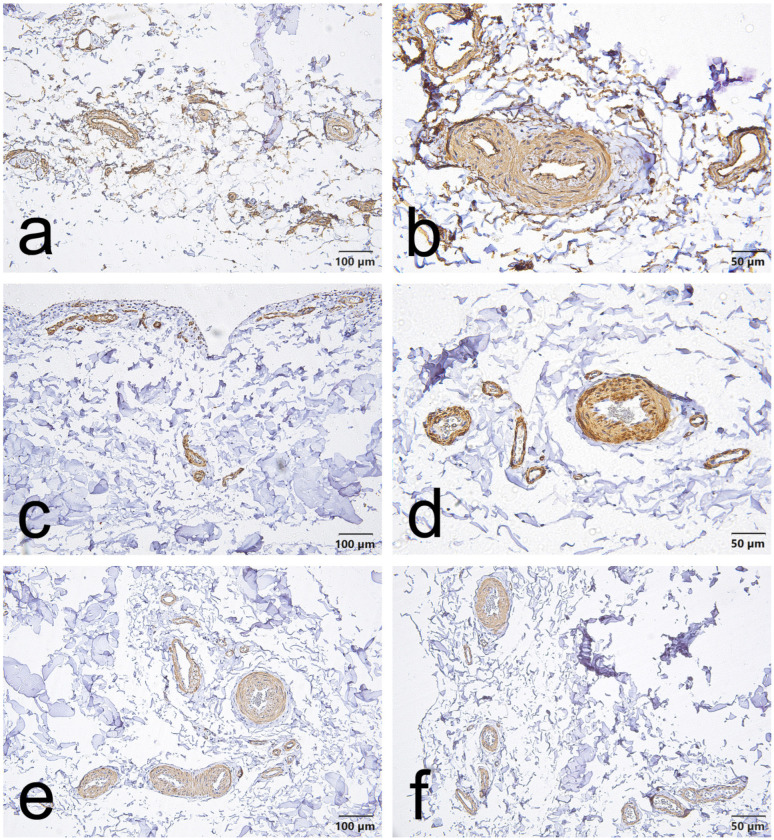
Representative immunohistochemistry of the proximal part of the MCL epiligament (EL). Panels show serial fields stained for CD34 (**a**,**b**), α–smooth muscle actin (α-SMA) (**c**,**d**), and vascular endothelial growth factor (VEGF) (**e**,**f**). Panels (**a**,**c**,**e**), scale bar = 100 µm; panels (**b**,**d**,**f**), scale bar = 50 µm.

**Figure 5:**
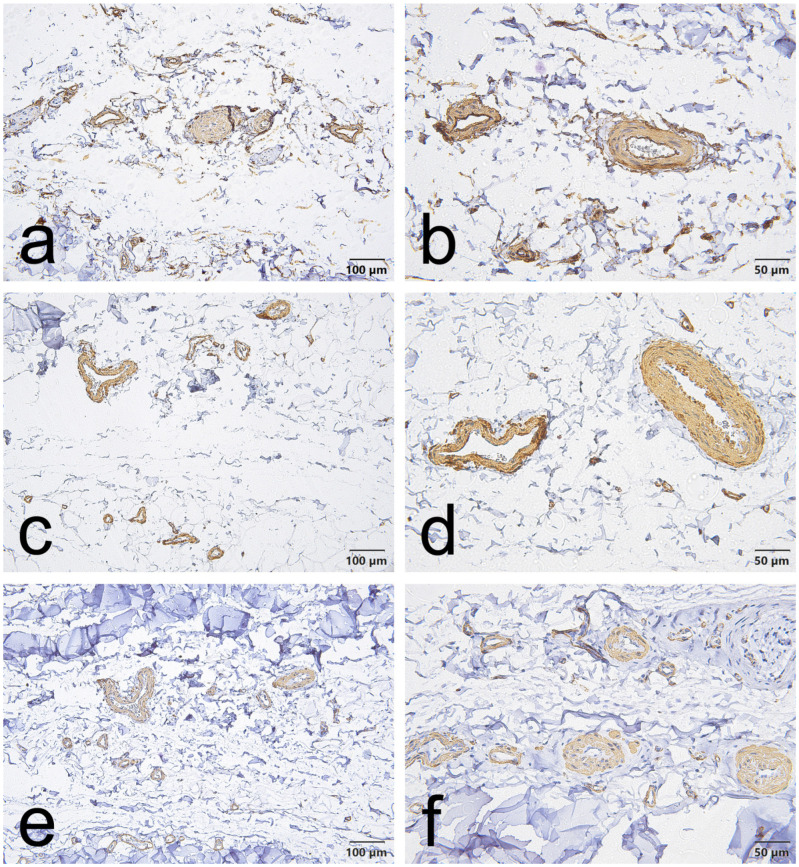
Representative immunohistochemistry of the mid-substance of the MCL epiligament (EL). Panels show serial fields stained for CD34 (**a**,**b**), α–smooth muscle actin (α-SMA) (**c**,**d**), and vascular endothelial growth factor (VEGF) (**e**,**f**). Panels (**a**,**c**,**e**), scale bar = 100 µm; panels (**b**,**d**,**f**), scale bar = 50 µm.

**Figure 6:**
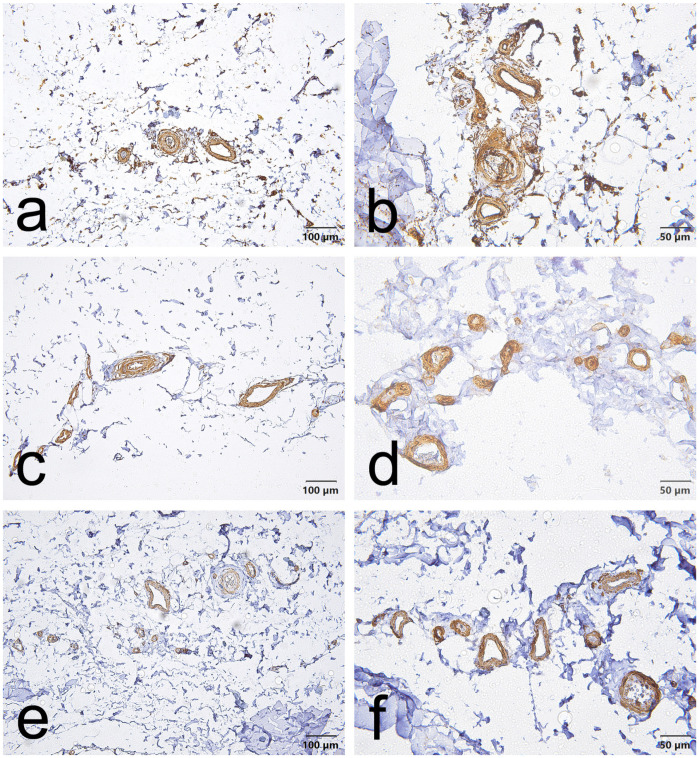
Representative immunohistochemistry of the distal part of the MCL epiligament (EL). Panels show serial fields stained for CD34 (**a**,**b**), α–smooth muscle actin (α-SMA) (**c**,**d**), and vascular endothelial growth factor (VEGF) (**e**,**f**). Panels (**a**,**c**,**e**), scale bar = 100 µm; panels (**b**,**d**,**f**), scale bar = 50 µm.

CD34, α-SMA and VEGF staining in the EL demonstrated consistent vascular localization but with region-dependent patterns that differed between the ACL and MCL. Overall, CD34 immunoreactivity was chiefly located in the endothelial lining of blood vessels in both ligaments. However, in the ACL, this endothelial CD34 signal was also diffusely evident throughout the proximal and distal EL (Figure 1a,b, Figure 2a,b and Figure 3a,b); in the MCL, CD34 was weak or absent in the endothelial layers of the proximal and distal EL and was more evident only in mid-substance vascular structures (Figure 4a,b, Figure 5a,b and Figure 6a,b).

α-SMA staining was predominantly confined to vascular smooth muscle within the tunica media in both ligaments. Notably, α-SMA was absent from the superficial EL layer of the ACL (Figure 1c,d, Figure 2c,d and Figure 3c,d) but was prominent in the MCL superficial EL, the latter showing more intense superficial and medial vascular staining than the mid-substance (Figure 4c,d, Figure 5c,d and Figure 6c,d).

VEGF immunoreactivity was likewise localized to vascular compartments: positive signals were primarily observed in vessel walls (including the tunica media) and endothelial layers, the ACL mid-substance displaying the most pronounced endothelial VEGF reactivity (Figure 3e,f). In contrast, VEGF was less apparent in the proximal and distal EL regions of both ligaments (Figure 1e,f, Figure 2e,f, Figure 3e,f, Figure 4e,f, Figure 5e,f and Figure 6e,f).

Taken together, these results indicate that although all three markers are concentrated around vascular structures, the ACL EL shows diffuse endothelial CD34 (especially proximally and distally) and no superficial α-SMA. In contrast, the MCL EL shows reduced proximal/distal CD34 but prominent superficial α-SMA. These findings demonstrate region-specific EL phenotypes that differ between the two ligaments.

### 3.1. Regional Heterogeneity in the ACL EL

Qualitative scoring of immunostained sections confirmed pronounced regional heterogeneity in marker expression across the ACL EL.

- For CD34, the proximal third of the ACL EL was predominantly low-positive (approximately 90% of fields), whereas the distal third exhibited the highest proportion of unequivocally positive fields (≈16%), consistent with an enriched vascular/progenitor signal at the tibial insertion.
- α-SMA showed a regionally variable pattern: the proximal EL was chiefly low-positive (≈66% of fields); the mid-substance displayed a roughly even distribution of negative and low-positive fields (each ≈ 40%) with the remainder positive (≈20%); the distal EL contained a moderate fraction of low-positive and positive fields (≈30% and ≈20%, respectively), such that ~50% of fields overall were negative.
- VEGF reactivity was absent in both proximal and distal regions (100% negative) but was concentrated in the mid-substance, which retained the largest proportion of low-positive fields (≈55%) and a substantive positive fraction (≈25%), indicating localized angiogenic activity.

### 3.2. Marker Expression in the MCL Epiligament (EL)

**Figure 7:**
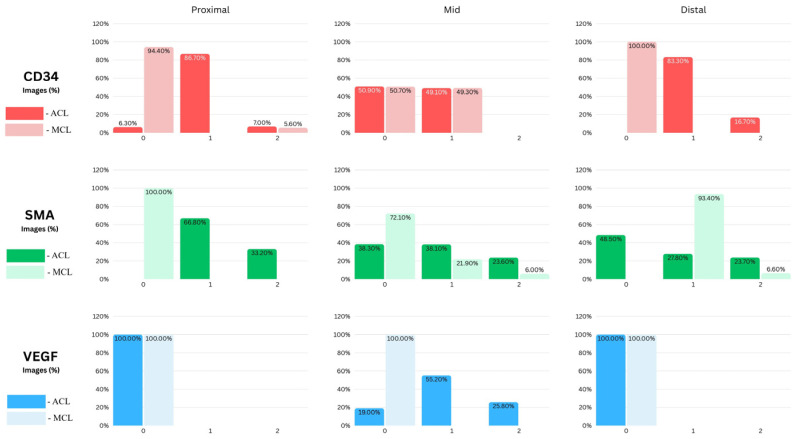
Comparative semiquantitative ImageJ/IHC Profiler analysis of CD34, α-SMA, and VEGF expression in the epiligament (EL) of the ACL and MCL, stratified by region (Proximal, Mid, Distal). Bars represent the percentage of images within each staining category (0 = negative, 1 = low-positive, 2 = positive; all groups sum to 100%).

In contrast to the ACL, the MCL EL showed a more uniform, consistently positive expression pattern across regions (Figure 7). CD34 was predominantly negative in the proximal (≈90%) and distal (100%) regions. For α-SMA staining, all proximal images (100%) had negative expressions, some fields in the mid-substance expressed low-positive (≈20%) and positive (≈6%) staining, but still most fields were negative (≈74%); whereas all distal images (100%) had low-positive expressions. VEGF remained uniformly negative throughout, with all proximal, mid, and distal fields showing consistent staining intensity (~100% negative).

### 3.3. Direct Comparison Between ACL and MCL ELs

Comparison of the EL of the ACL and MCL revealed clear region-specific differences. In the proximal EL, the ACL showed markedly greater CD34 and α-SMA immunoreactivity than the MCL (both *p* < 0.0001). VEGF was absent from both proximal ELs. In the mid EL, CD34 expression was comparable between ligaments, whereas α-SMA was significantly higher in the ACL (*p* < 0.0001); VEGF was present in the ACL mid EL but absent from the MCL mid EL (*p* < 0.001). In the distal EL, CD34 and α-SMA distributions again differed between ACL and MCL (CD34 *p* < 0.0001; α-SMA *p* < 0.001), while VEGF was absent from both distal ELs. The underlying data are shown in Figure 7 and summarized in Table 1.

Overall, the ACL EL exhibits higher endothelial (CD34) and myofibroblastic (α-SMA, VEGF at mid) expression than the MCL at proximal and mid sites, the most striking contrasts being in the proximal and distal regions. These results are summarized in Table 1.

## 4. Discussion

The present study provides a detailed, region-specific comparison of EL morphology between the ACL and MCL. It links those molecular and cellular maps to established clinical differences in reparative capacity. Our immunohistochemical and image-analytic data show an apparent dichotomy: the ACL EL is regionally heterogeneous and vascular-biased, whereas the MCL EL is comparatively uniform in marker distribution and connective-tissue–oriented. These differences, manifest in CD34, α-SMA, and VEGF expression patterns, cell densities, and marker localization, offer a mechanistic bridge between EL biology and the long-observed ACL–MCL healing paradox. Three principal findings emerge from our study:

- First, the EL of the ACL displays marked regional heterogeneity. The proximal ACL EL was dominated by low-positive CD34 staining with a small positive fraction, whereas the distal ACL contained the highest proportion of CD34-positive fields; VEGF was absent from both proximal and distal sites. α-SMA expression in the ACL was most prominent proximally and showed a mixed profile in the mid-substance.
- Second, the EL of the MCL is relatively homogeneous but follows a pattern distinct from the ACL. Proximal MCL EL fields were essentially negative for α-SMA, whereas the distal MCL EL was dominated by low-positive α-SMA staining; VEGF reactivity was uniformly absent across all MCL regions.
- Third, segmentation and comparative analyses revealed clear, statistically robust contrasts between the ligaments. Differences in proximal and distal CD34 and α-SMA between ACL and MCL were highly significant (proximal CD34/α-SMA and distal CD34, *p* < 0.0001; distal α-SMA, *p* < 0.001), while mid-substance CD34 distributions did not differ (*p* = 0.998). The mid ACL alone retained a measurable VEGF signal, whereas the mid MCL showed no VEGF reactivity (mid ACL vs. mid MCL, *p* < 0.001).

Taken together, these results define discrete, region-specific EL “signatures.” The MCL EL pattern—particularly distal low-positive α-SMA—appears well suited to support wound contraction and local tissue reconstitution, whereas the ACL EL exhibits patchy, vascular-centered reparative signals that may not promote organ-level healing.

The high incidence of α-SMA–positive cells in the EL of the MCL supports a prominent role for myofibroblasts in MCL repair. These findings are consistent with and extend previous work on ligament biology: the MCL’s superior healing capacity has been attributed to its extra-articular location, rich vascularity, and favorable mechanical environment [5,6,18]. The α-SMA distribution we report—in particular the predominance of low-positive α-SMA staining in distal MCL fields—provides a molecular correlate for myofibroblast-mediated contraction, despite the absence of detectable VEGF in the MCL EL in our series. The high distal cell density reported in the Results, together with distal α-SMA predominance, helps explain why distal regions of the MCL, when not mechanically entrapped, exhibit greater healing capacity: they are able to contract efficiently and reconstitute tissue continuity. By contrast, the EL of the ACL shows a more superficial α-SMA layer that is most pronounced proximally and present to a lesser degree mid- and distally; this spatial pattern may promote maladaptive shortening and remnant retraction, thereby undermining effective matrix bridging. Our observations of α-SMA distribution are in line with graft-remodeling studies by Weiler et al., which demonstrated myofibroblast involvement in early fiber formation and their persistence during ACL graft maturation [16,32]. The regionally distinct α-SMA patterns described here therefore provide a plausible substrate for the observed differences in matrix architecture and mechanical behavior of repaired ACL versus MCL tissue [32].

However, the ACL presents a more complex picture. The historical failure of primary ACL repair and the widespread adoption of reconstruction were founded on observations of poor spontaneous mid-substance healing [10]. Our data illuminate several mechanistic contributors. Although the ACL EL contained measurable CD34 signals (notably low positive/positive proximally and distally) and a mid-substance VEGF signal (mid ACL: ≈55.2% low positive/25.8% positive), the overall pattern was regionally patchy and biased toward vascular compartments rather than being diffusely distributed through the connective tissue. This vascular location suggests a reservoir of progenitor potential but limited tissue-wide delivery or engagement. The incidence of α-SMA–positive cells in the ACL EL observed in our study highlights a potentially important role for myofibroblast-like activity in ACL function and repair. Murray and Spector [40] previously described myofibroblast-like cells expressing α-SMA within the midsubstance of the ACL, and in a subsequent study Murray et al. [41] proposed that retraction of ACL remnants may result from a continuous α-SMA–positive layer rather than isolated myofibroblasts. Our finding of superficial α-SMA immunoreactivity in the mid-substance ACL EL is concordant with Murray et al.’s observations [41]. It is important to note, however, that Murray et al. [41] discussed EL tissue together with synovial tissue in their characterization. In contrast, our prior work defined the ACL EL specifically as the tissue immediately surrounding the ligament itself [18,38,39]. Although the EL is morphologically similar to synovium, it is best regarded as a specialized variant of synovium with distinct structural and functional properties [42]. This distinction should be borne in mind when comparing immunohistochemical patterns and inferring mechanisms of remnant retraction or repair. Notably, the pronounced α-SMA reactivity observed in the ACL’s superficial EL, most prominent proximally, suggests a contractile sheath that, when activated, could drive remnant retraction rather than gap closure. In other words, an α-SMA-rich superficial EL could function as a “retraction engine,” shortening and pulling stumps away from each other and thereby preventing effective bridging, particularly in mid-substance ruptures. This provides a molecular explanation for the clinical observation that proximal avulsion tears (which preserve remnant tissue and lack this deleterious superficial contractile arrangement) are more amenable to repair [43,44].

The CD34 findings deserve particular emphasis. Preclinical studies show that CD34+ endothelial progenitors augment neovascularization, collagen synthesis, and tendon–bone integration [26,45,46,47]. Our data show that ACL EL images contain a higher overall proportion of low-positive and positive CD34 scores at proximal and distal sites than MCL EL (where proximal and distal CD34 were predominantly negative). This suggests that progenitor cells are present within ACL remnants and EL, consistent with prior evidence that vascular walls serve as reservoirs of stem and progenitor cells [48,49]. Matsumoto et al. [47] reported that CD34-expressing vascular cells can differentiate into multiple lineages and migrate to the site of ACL rupture, potentially contributing to repair [27]. Mifune and colleagues further demonstrated that transplantation of CD34+ endothelial progenitor cells derived from human ACL remnants enhances tendon–bone healing in a rat ACL-rupture model [45], and that ruptured ACL remnants contain a higher abundance of vascular-derived CD34+ cells than uninjured midsubstance tissue [46]. Our findings are concordant with these reports and with Kirizuki et al.’s conclusions regarding the benefits of remnant-preserving ACL surgery [50]. Specifically, we observed hypercellularity of the ACL EL and focal enrichment of CD34+ cells, which plausibly support improved graft incorporation and healing. Moreover, the preferential expression of CD34+ cells in the proximal and distal ELs, together with greater cellularity at these sites, may facilitate healing at the ligament ends relative to the midsubstance—an observation that aligns with clinical data [51,52,53,54].

The expression of VEGF further underscores the tissue-specific differences but deviates from some prior expectations. In our samples, VEGF immunoreactivity was restricted to the mid part of the ACL (mid ACL: ≈55.2% low positive/25.8% positive). It was absent proximally and distally in both ligaments, and absent in all MCL regions (100% negative). VEGF is a central driver of angiogenesis [55,56,57] and peaks early during proliferative repair [27,35,58]. The presence of VEGF in the ACL mid-substance suggests a focal angiogenic competence that is perhaps insufficiently distributed to support whole-organ healing, whereas the absence of detectable VEGF in the MCL EL in our cohort indicates that EL-mediated VEGF signaling is not the sole explanatory mechanism for the MCL’s superior healing in this dataset. Experimental models demonstrating VEGF’s beneficial effects on MCL repair [27] remain relevant, but our data imply that VEGF dynamics in human ELs are regionally constrained and that other factors (myofibroblast distribution, mechanical milieu, extrinsic vascular access) are likely to be critical determinants of outcome [58,59]. Matsubara et al. [60] reported a correlation between increased VEGF expression and degenerative changes in the ACL in osteoarthritic knees and noted that ACLs from osteoarthritis patients exhibit higher VEGF levels than those from trauma patients. Pufe et al. [61] similarly observed that VEGF is minimally expressed in healthy tendons but increases with degeneration. If molecular and morphological features are shared between tendons and ligaments [62,63], the focal VEGF immunoreactivity we observed predominantly in the midportion of the ACL EL may reflect incipient degenerative change at that site and could contribute to the higher incidence of injury reported in this region. These data are correlative, however, and do not prove causation; longitudinal and mechanistic studies will be required to determine whether elevated VEGF expression promotes degeneration or is a secondary response to tissue damage.

### 4.1. Limitations

Several limitations of the present study merit explicit acknowledgement:

- First, the immunohistochemical readouts reported here are semi-quantitative and therefore do not constitute direct measures of absolute protein abundance or of functional activity in vivo. We used an automated ImageJ/IHC Profiler pipeline to reduce subjective thresholding and operator bias; however, the ordinal intensity categories generated by this approach (for example “low-positive” versus “positive”) are relative measures that do not scale linearly with molecular concentration. Interpretation of biological effect therefore requires caution and, where possible, corroboration by complementary quantitative methods or functional assays.
- Second, although automated scoring reduces manual subjectivity, field selection and region delimitation remain potential sources of variability. Regions of interest were defined according to anatomical landmarks and applied consistently, but assignment of boundaries and the choice of visual fields can influence measured outcomes. We did not perform formal inter- or intra-observer reliability testing (ICC/κ) for region assignment in the present dataset; this omission is acknowledged as a limitation and should be addressed in future work.
- Third, we took steps to minimize technical variability (slides processed in a single staining batch and standardized image acquisition), but residual technical confounders—DAB development variability, subtle exposure/white-balance differences, and unrecognized batch effects—cannot be ruled out entirely. These factors can affect apparent staining intensity and therefore the semi-quantitative classifications derived from images.
- Fourth, the statistical inferences are constrained by sample size and the exploratory nature of some comparisons. The present sample provides robust within-study contrasts for primary endpoints but limits the ability to perform well-powered subgroup or age-stratified analyses. The sample size and retrospective imaging analyses limit subgroup or age-stratified conclusions (for instance, differences in remnant CD34 content with age noted by others [57] could not be fully explored). In addition, multiple regional and marker comparisons increase the risk of type I error; readers should therefore interpret isolated marginal *p*-values cautiously unless supported by effect sizes and biological consistency. Future studies should pre-specify primary endpoints and apply appropriate multiplicity controls.
- Finally, histological compartmentalization is complex and our segmentation strategy (vascular versus connective tissue compartments) may not capture all micro-environmental interactions that influence marker expression. Complementary approaches—lineage or functional assays, ultrastructural analysis, and prospectively planned reliability assessments—would strengthen causal inference and better resolve the biological meaning of semi-quantitative IHC signals.

### 4.2. Future Directions

Future work should test the causal role of EL phenotypes in healing using interventional models: selectively augmenting EL CD34 pools, suppressing superficial α-SMA contractility, or locally delivering VEGF in a controlled, regionally targeted fashion would help to determine which manipulations can convert ACL mid-substance tears from non-healing to healing states. Translational studies to optimize cell source, dose, priming, and delivery vehicles (including decellularized EL-mimetic scaffolds or TDSC sheets) are indicated and guided by the EL signatures described here. Finally, clinical trials that stratify ACL injuries by EL phenotype (or at least by tear location and remnant quality) could clarify which patients can safely undergo non-operative management or remnant-preserving repair.

## 5. Conclusions

This study provides the first region-specific, quantitative comparison of the EL between the ACL and MCL, revealing clear molecular and structural distinctions that align with their contrasting healing capacities. The ACL EL demonstrated pronounced regional heterogeneity, with strong proximal α-SMA expression, mid-substance VEGF activity, and distal CD34 positivity, patterns indicative of localized but poorly integrated reparative potential. In contrast, the MCL EL was more uniform, displaying consistent connective-tissue distribution of α-SMA and largely negative CD34 and VEGF expression, reflecting a stable yet functionally efficient reparative architecture. Statistically significant differences in proximal and distal CD34 and α-SMA expression between the two ligaments underscore a mechanistic divergence: the ACL’s EL appears more vascularly biased and contractile, while the MCL’s EL is structurally organized to support tissue continuity and mechanical restoration.

Together, these findings suggest that the ACL’s failure to heal is not attributable to a lack of progenitor or angiogenic signals but to its fragmented spatial organization and a dominant superficial contractile phenotype. Conversely, the MCL’s coordinated α-SMA distribution and favorable extracellular environment facilitate effective repair despite limited VEGF activity. These insights reinforce the concept that successful ligament healing depends on the spatial balance between progenitor availability, angiogenic signaling, and controlled myofibroblast activity. Preservation and modulation of the EL, particularly its CD34+ and α-SMA+ compartments, should therefore be central to future biological and surgical strategies aimed at enhancing intrinsic ACL repair.

## Figures and Tables

**Table 1:** Summary Table depicting the expression of the assessed markers across the three parts of the EL of the ACL and MCL.

Marker	Region	ACL (Neg/Low Pos/Pos)	MCL (Neg/Low Pos/Pos)	*p*-Value
CD34	Proximal	6.30%/86.70%/7.00%	94.40%/0.00%/5.60%	<0.0001
	Mid	50.90%/49.10%/0.00%	50.70%/49.30%/0.00%	0.998
	Distal	0.00%/83.30%/16.70%	100.00%/0.00%/0.00%	<0.0001
α-SMA	Proximal	0.00%/66.80%/33.20%	100.00%/0.00%/0.00%	<0.0001
	Mid	38.30%/38.10%/23.60%	72.10%/21.90%/6.00%	<0.0001
	Distal	48.50%/27.80%/23.70%	0.00%/93.40%/6.60%	<0.001
VEGF	Proximal	100.00%/0.00%/0.00%	100.00%/0.00%/0.00%	1
	Mid	19.00%/55.20%/25.80%	100.00%/0.00%/0.00%	<0.001
	Distal	100.00%/0.00%/0.00%	100.00%/0.00%/0.00%	1

## Data Availability

The raw data supporting the conclusions of this article will be made available by the authors upon request.

## Abbreviations

The following abbreviations are used in this manuscript:

ACL	Anterior cruciate ligament
AM	Anteromedial (bundle of the ACL)
DAB	3,3′-diaminobenzidine
DOV	Deep MCL—meniscofemoral & meniscotibial components (contextually used)
EL	Epiligament
EPC	Endothelial progenitor cell
EPCs	Endothelial progenitor cells
FLEX	EnVision™ FLEX+ detection system
IHC	Immunohistochemistry
L-PRF	Leukocyte- and platelet-rich fibrin
MCL	Medial collateral ligament
MSC	Mesenchymal stem cell
MSCs	Mesenchymal stem cells
NIH	National Institutes of Health (ImageJ)
PCL	Posterior cruciate ligament (appears in comparative anatomical text)
PL	Posterolateral (bundle of ACL)
POL	Posterior oblique ligament
R	Statistical software R
RCT	Randomized controlled trial (appears in cited studies)
SMA/α-SMA	Alpha-smooth muscle actin
TDSC	Tendon-derived stem cell
TLA	Three-letter acronym
VEGF	Vascular endothelial growth factor
